# How I Teach Mechanical Ventilator Waveform Analysis: A Focus on Normal Ventilator Waveforms

**DOI:** 10.1093/atsscholar/aapag020

**Published:** 2026-04-20

**Authors:** Michael Keller, Nitin Seam, Burton Lee

**Affiliations:** Division of Pulmonary and Critical Care Medicine, University of Maryland School of Medicine, Baltimore, MD, United States; Section of Pulmonary and Critical Care Medicine, Baltimore Veterans Affair Medical Center, Baltimore, MD, United States; Division of Pulmonary and Critical Care Medicine, University of Maryland School of Medicine, Baltimore, MD, United States; Section of Pulmonary and Critical Care Medicine, Baltimore Veterans Affair Medical Center, Baltimore, MD, United States; Department of Critical Care Medicine, National Institutes of Health Clinical Center, Bethesda, MD, United States

**Keywords:** mechanical ventilation, waveform analysis, respiratory failure, medical education, asynchrony

## Abstract

Identification and management of patient-ventilator asynchronies is a fundamental skill for the management of mechanical ventilation and often requires interpretation of mechanical ventilation waveform scalars. However, teaching mechanical ventilation waveform analysis can be challenging for educators due to the complexity of the material. In this entry of How I Teach, we provide an approach to teaching the concept of “normal” mechanical ventilator waveforms as an introduction to the broader topic of patient-ventilator asynchronies. We stress that intimate knowledge of normal mechanical ventilator appearance and physiology is vital prior to tackling the concepts of abnormal mechanical ventilator waveforms. We offer a framework to present this material in a classroom or small group setting, summarize key concepts, and provide concrete examples of the educational techniques we utilize.

## Introduction

Patient-ventilator asynchrony (PVA) is a common and frequently unrecognized problem in the intensive care unit (ICU) that results in serious clinical consequences.^1-3^ Clinicians caring for patients on mechanical ventilation (MV) should have a basic understanding of how to identify and address serious and life-threatening PVAs. In fact, current competency expectations for fellowship training in pulmonary and critical care medicine specifically include the milestone of “management and direction of the use of mechanical ventilation.”^4^ Contemporary approaches to teaching MV emphasize concepts relating to the basic modes of ventilation, goals of gas exchange, and avoidance of ventilator-associated lung injury, yet some fundamental concepts are not commonly taught due to the need to incorporate complicated physiologic principles. Even when educators possess a thorough understanding of the principles pertaining to the identification of MV waveform asynchronies, effectively conveying these complex concepts to learners can be challenging. Beginning with normal, passive MV waveforms provides a framework for teaching and learning the appearance of MV waveforms and the physiological principles before advancing to abnormal ventilator waveforms and PVAs.

What makes teaching normal passive MV waveforms challenging? First, trainees may lack the requisite knowledge of the mathematical and physiological principles that dictate the relationship between airway pressure (P_aw_), flow (F), tidal volume (TV), and respiratory system mechanics. Educators may also face challenges with presenting certain physiologic concepts in a manner that is practical and clinically relevant for the trainee, especially given the time constraints present in the ICU. Furthermore, there has been a lack of standardization in how MV is taught, with significant heterogeneity in educational content and absence of a standard educational framework.^5^

This How I Teach article presents our approach to teaching normal MV waveforms to fellows and early career faculty working in the ICU. While we introduce these lessons first in a large-scale course of PVA, here we provide a framework for educators to effectively present the concepts of normal MV waveforms to learners, either in the classroom or small group setting.

## Who are the learners?

This article is aimed at experienced fellows and attendings who hope to teach normal MV waveforms to junior fellows. However, other trainees and staff with significant background or exposure to more advanced concepts in MV may be appropriate, including senior residents, respiratory therapists, and advanced practice providers. While some of the concepts presented here are appropriate for medical students and residents, the complexity and focus of the material makes this best suited for fellows and faculty.

## What is the setting?

Our preferred approach involves presenting the material in a classroom setting to fellows and attendings as part of a multi-day small-group and simulation-based education course,^6^ but the material presented here can be divided into several blocks and gradually presented in 15- to 30-minute teaching sessions. These concepts are most suitably presented in a classroom setting but can also be incrementally taught prior to rounds, supplemented by beside chalk talks, or reviewed with real-world, practical examples.

## What is the content?

For the purposes of this review, we will focus on normal waveforms and omit other key concepts that we traditionally cover, including the identification, consequences, and management of specific PVAs. In this regard, our content specifically focuses on an intimate understanding of the equation of motion and how the components of this equation determine normal MV waveforms. We primarily focus on inspiration, as expiration has been covered in a previous How I Teach article.^7^

## What is the approach?

We approach the teaching of complex topics in MV by chunking the material into smaller sections and presenting learners with a series of manageable questions that gradually build on each other. This approach promotes learner participation, gives proper context for the topic, and offers the educator a basic framework for presenting the subject in a logical, well-organized manner. We first concentrate on the principles of Ohm’s Law and the equation of motion before tying these equations together with the structure of normal MV waveforms.

### Presenting the topic: the equation of motion

We start with the concept of Ohm’s law and its derivative, the equation of motion—a concept fundamental to understanding the principles of MV. In our experience, learners have considerable trouble understanding the subsequent topics without an intimate understanding of the equation of motion. Explaining the basic concepts of the equation of motion typically takes approximately 1 hour, however, in the setting of time constraints, one may divide the topic into 30-minute blocks (such as one session describing the derivation of the equation and the second session walking through the corresponding exercises described in the supplementary index below).

**Figure 1:**
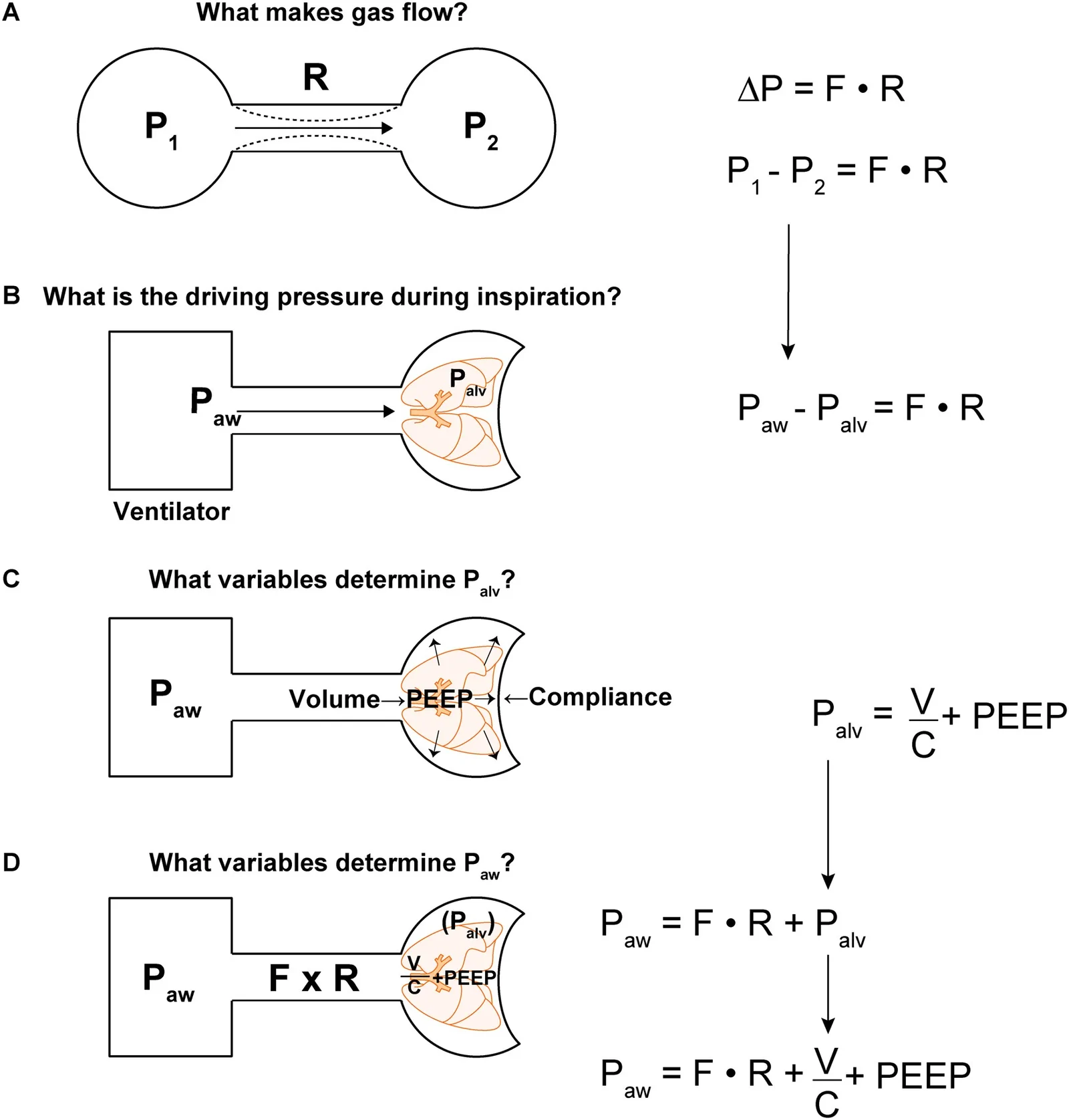
A step-by-step framework for introducing the derivation of the equation of motion from Ohm’s law using a “balloon and tube” model as a guide. (A) Balloon and tube model demonstrating 2 compartments connected by a tube and the pressure of each compartment labeled as P_1_ and P_2_. We emphasize that, according to Ohm’s law, a pressure gradient must be present for airflow to occur. Resistance is the variable that makes it difficult for gas to flow and is primarily determined by the diameter of the tube. (B) To translate the concept of Ohm’s Law to mechanical ventilation, we convert P_1_ and P_2_ to P_aw_ and P_alv_, respectively, forming the pressure gradient for flow during inspiration. (C) We emphasize that P_alv_ is determined by 3 key variables: volume, compliance, and PEEP. (D) Rearranging our equation from B and replacing P_alv_ with its component variables from C, we now have the final derivation of the equation of motion, which states that P_aw_ at any given time is determined by 5 key variables: flow, resistance, volume, compliance, and PEEP. Abbreviations: C, compliance; F, flow; P_alv_, alveolar pressure; P_aw_, airway pressure; PEEP, positive end expiratory pressure R, resistance; V, volume.

We start by drawing a tube connected to 2 balloons on each side, asking, “What makes a gas flow across the tube from one balloon to the other?” (Figure 1A) We next label each balloon with either “P_1_” or “P_2_” to designate the pressures that form the pressure gradient for flow (F), drawing an arrow from the balloon with higher pressure to the balloon with lower pressure in the direction of F. We then ask, “What makes it difficult for a gas or a liquid to flow?” labeling the tube with an “R” to indicate resistance (R) and thereby displaying the completed Ohm’s law equation next to the figure. We then ask, “If there is little flow between the balloons, what may be inferred about the pressures in the two balloons or about the resistance of the tubing?” to emphasize that a lack of F results when there is no longer a pressure gradient or there is high R. These questions introduce the primary elements of Ohm’s law that govern airflow:


Pressure Difference (ΔP)=Flow (F) x Resistance (R) and thus, F=ΔP / R.

Next, we ask learners to imagine that balloon P_1_ is the ventilator and label it as P_aw_—the proximal airway pressure near the ventilator. We then designate balloon P_2_ as the alveoli and label it as P_alv _(alveolar pressure). We now emphasize, that in a mechanically ventilated patient, the driving pressure for F (ΔP) can be conceptualized as the difference between P_aw_ and P_alv_ (Figure 1B). Therefore, with positive pressure ventilation, airflow occurs during inspiration when a pressure gradient is generated between the ventilator (P_aw_) and the patient (P_alv_) such that our original Ohm’s law equation now becomes:


$$P_{aw}–\;P_{\mathrm{alv}}=F\;x\;R.$$

We now turn our attention to the physiologic components that make up P_alv_. We ask, “What would cause the pressure in the P_alv_ balloon to change?” We emphasize that P_alv_ is dependent on the change in volume (V) in the alveoli, respiratory system compliance (C), and the total positive end expiratory pressure (PEEP_total_; Figure 1C) such that:


$$P_{\mathrm{alv}}=V/C+\mathrm{PEE}P_{\mathrm{total}}.$$

We present the topic of C by first asking, “What determines the pressure in a given balloon?” Because C is the ratio of the change in volume (ΔV) over change in pressure (ΔP), or C = ΔV / ΔP, the learners will realize that pressure will be higher with larger tidal volumes (V_T_) or with lower C. Further, questions such as “What would happen to pressure in the balloon as it fills with more volume?” or “How would pressure in the balloon change if the balloon was ‘stiffer’ or ‘floppier’?” solidify these concepts.

Our Ohm’s law equation can now be further rearranged to illustrate each of the components that make up P_aw_ (Figure 1D):


$$P_{aw}=F\;x\;R+V/C+\mathrm{PEE}P_{\mathrm{total}}.$$

This equation is known as the equation of motion and is fundamental to the understanding of MV. We stress that it is simply an extension of Ohm’s law and states that the P_aw_ at any given time is determined by the product of F x R plus the components of P_alv_ (V/C + PEEP_total_). We use the equation of motion to further explain why ventilator waveforms appear as they do and how they are affected by changes in respiratory system mechanics and patient-ventilator interactions.

Finally, we emphasize 3 key pressures described by the equation of motion: (1) the peak inspiratory pressure (PIP; the highest P_aw_ over the course of inspiration); (2) the plateau pressure (P_plat_; the P_alv_ at the end of inspiration during an inspiratory pause maneuver); and (3) the end expiratory hold pressure (PEEP_total_; P_alv_ at the end of expiration during an expiratory pause maneuver). To further underscore these concepts using the equation of motion, we ask, “What is the difference between the PIP and P_plat_?” Scanning the equation of motion allows the learners to realize that because PIP is the highest P_aw_ over the course of inspiration (during flow delivery), it is dictated by all 5 variables of the equation of motion (F, R, V, C, and PEEP_total_). However, because P_plat_ represents the alveolar pressure at the end of inspiration during an inspiratory pause (where flow delivery is zero), the F x R component of the equation cancels out, and P_alv_ is dictated by V, C, and PEEP_total_, but not F or R (Figure S1). With these variables in mind, the learner now has a thorough understanding of what dictates the various clinically relevant pressures recorded on the mechanical ventilator but also allows for the systematic formulation of a differential diagnosis in the setting of changes of each of these pressures by simply referring to the variables involved (Figure S2).

With an introduction of the equation of motion in hand, we move on to highlighting how the variables of the equation of motion influence various ventilator modes and their corresponding normal, passive waveforms.

### Presenting the topic: normal waveforms

Our approach emphasizes that to understand abnormalities that may arise in a mechanically ventilated patient, the learner should first understand how normal, passive MV waveforms look. We define *normal* as the absence of significant alterations in respiratory system mechanics, pathology or significant patient interaction. Depending on the setting, we typically spend 3 approximately 1-hour lectures dedicated to this topic, with each lecture dedicated to a specific mode of MV (again, these lectures may also be split into 15- to 30-minute chunks, for instance, one describing the appearance of the waveforms and another session dedicated to having the learners draw the waveforms). We typically cover volume control ventilation (VCV; both square and decelerating flow patterns), pressure control ventilation (PCV), and volume-targeted ventilation (VTV). To save lecture time, educators could consider focusing on the most common mode of ventilation used in their ICU.

We teach that, by convention, the most commonly displayed waveforms on a mechanical ventilator are the airway pressure-time (P_aw_-time), flow-time (F-time), and volume-time waveforms (V-time). Our approach begins by asking a series of questions that learners should systematically ask themselves each time they approach interpretation of waveforms:

1. Which curve(s) is fixed (ie, controlled by the clinician)? We point out that the answer to this question depends on the “type” of breath being delivered. Emphasizing the equation of motion, we explain that one may either set the P_aw_ as in PCV, or the V and F as in VCV. We emphasize that the area under the inspiratory F-time curve is tidal volume (V_T_) and the slope of the V-time curve is F, which means that V and F are intimately related. Therefore, if the mode controls V, then F is also being controlled, and if F is fixed, V is also fixed. Starting with which curve is fixed allows one to identify the most predictable curve (ie, the curve that is set by the clinician on the ventilator and should appear relatively unchanged from breath to breath).
2. Which curve(s) is variable? Once we know which curve is fixed, we can now ask which curve(s) is variable based on the equation of motion. For PCV, we explain that because the P_aw_-time curve is fixed, the V-time and F-time curves are variable. For VCV, the opposite is true, whereby the clinician sets V and F, and the P_aw_-time curve is variable. It is important for learners to recognize which curve is variable because, in general, the variable curve will change in response to physiologic alterations, pathology, and patient-ventilator interaction.^8^
3. What should the curve look like? It is vital to understand approximately how a normal passive ventilator waveform should appear. Once a mental model is made of what a normal waveform should look like in various modes, we can then compare to the actual waveforms being displayed to identify pathology or asynchronies.

As we ask these questions, we guide the learners through a table like Table 1. In addition, we may have the learners write out the equation of motion and circle which components are fixed, which are patient characteristics (eg, R, C), and which are variable for each mode of MV (Table 2).

**Table 1:** Fixed and variable components of the different modes of mechanical ventilation and what equation dictates the variable component.

	Pressure control		Volume control		Volume targeted	
	Inspiration	Expiration	Inspiration	Expiration	Inspiration	Expiration
**Pressure**	Fixed	Fixed	Equation of motion	Fixed	Variable	Fixed
**Flow and volume**	Ohm’s	Ohm’s and natural decay	Fixed	Ohm’s and natural decay	Variable	Ohm’s and natural decay

**Table 2:** The relationship of components in the equation of motion with different modes of mechanical ventilation.

	Volume control	Pressure control	Volume-targeted
**Clinician controlled?**	F, V, PEEP_set_	P_aw,_ PEEP_set_	PEEP_set_
**Patient characteristics?**	R, C, P_musc_	R, C, P_musc_	R, C, P_musc_
**Variable?**	P_aw_	F, V	F, V, P_aw_*
**Full equation**	P_aw_−P_musc_ = F * R + V/C + PEEP_set_	P_aw_−P_musc_ = F * R + V/C + PEEP_set_	P_aw_−P_musc_ = F * R + V/C + PEEP_set_

*In volume-targeted ventilation, Paw varies as the ventilator adjusts inspiratory pressure to achieve the target tidal volume.

Abbreviations: C, compliance; F, flow; P_aw_, airway pressure; Pmusc, pressure generated by respiratory muscles; _PEEP_, positive-end expiratory pressure; R, resistance; PEEP_set_, PEEP set by the clinician; V, volume.

With an understanding of how we systematically approach waveform analysis with respect to the equation of motion, we now tackle how to “build” normal waveforms for each mode of MV.

### Volume control breath—square-wave flow ventilation

We begin by reiterating the questions above: Which curve(s) is fixed for VCV? For VC square-waveform ventilation, we emphasize that the fixed curves during inspiration are V-time and F-time curves and should not vary substantially from breath to breath. Therefore, the inspiratory F-time curve can be drawn as a rectangular pattern since the flow is constant. Furthermore, the V-time curve can be drawn as a linear pattern, since the slope of the V-time curve is F, and F is constant in a square-wave flow pattern.

**Figure 2:**
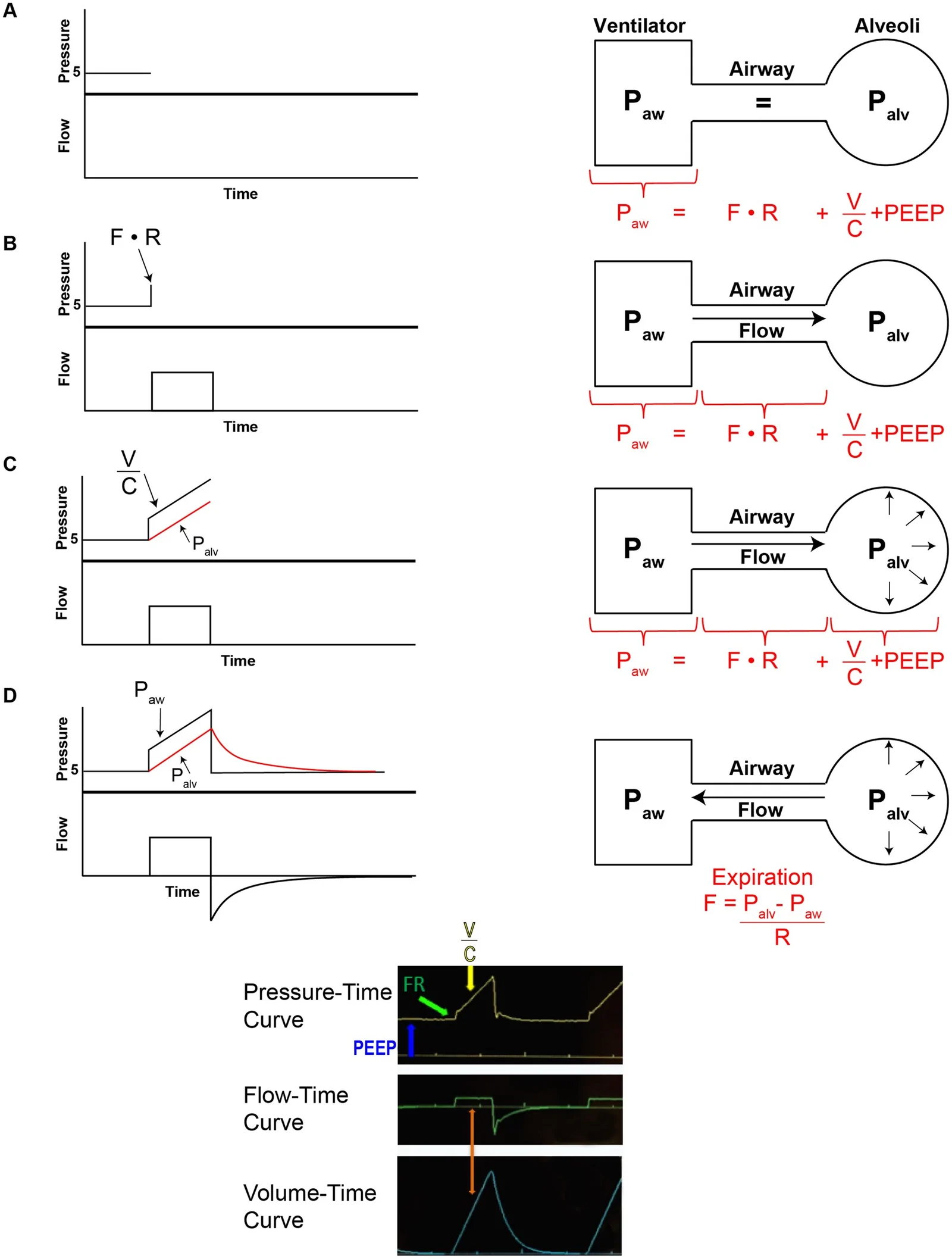
A step-by-step approach to teaching how the normal VCV square waveform is constructed focusing on its relationship to the equation of motion. (A) During expiration, the P_aw_ is set by the clinician as PEEP_set_ and appears as a horizontal line. (B) At the start of inspiration, the ventilator rapidly increases P_aw_ to generate inspiratory F, and F is delivered in a constant, square-wave fashion. This appears as a vertical line on the P_aw_-time curve and represents the F x R component of the equation of motion. (C) As air fills the alveoli, and assuming C remains constant throughout inspiration, V will increase in a linear fashion (given the constant F), and therefore, P_alv_ and P_aw_ will also increase in a linear fashion. Because F is a constant value throughout inspiration, the difference between P_aw_ and P_alv_ will remain constant throughout the course of inspiration. The slope of this portion of the P_aw_-time curve is determined by V/C. (D) Once the set TV has been delivered, the ventilator will transition from inspiration to expiration. The gradient for F now reverses and is directed from the patient toward the ventilator. Abbreviations: C, compliance; F, flow; P_alv_, alveolar pressure; P_aw_, airway pressure; PEEP, positive end expiratory pressure; R, resistance; TV, tidal volume; V, volume; VCV, volume control ventilation.

We then ask, “Which curve(s) is variable for VCV?” Because the F-time and V-time curves are fixed in VCV, the P_aw_-time curve will be variable and can be modeled according to the equation of motion. As we draw the P_aw_-time curves, we draw arrows pointing to the parts of the curve that are dictated by their corresponding components in the equation of motion. Emphasizing that these are passive breaths, we draw each part of the curve as we teach the following: just before the start of inspiration, the P_aw_ is the PEEP set by the clinician (PEEP_set_), represented by a horizontal straight line at the level of PEEP_set_ during the expiration (Figure 2A). Next, as the ventilator generates the set F and overcomes R of the airways, P_aw_ rapidly increases at the start of inspiration in the form of a vertical line (Figure 2B). We stress that the height of this line is represented by the F*R component of the equation of motion. Thus, higher set F or R will increase the height of this vertical line as higher P_aw_ will be needed to achieve a higher flow rate or overcome a higher R.

**Figure 3:**
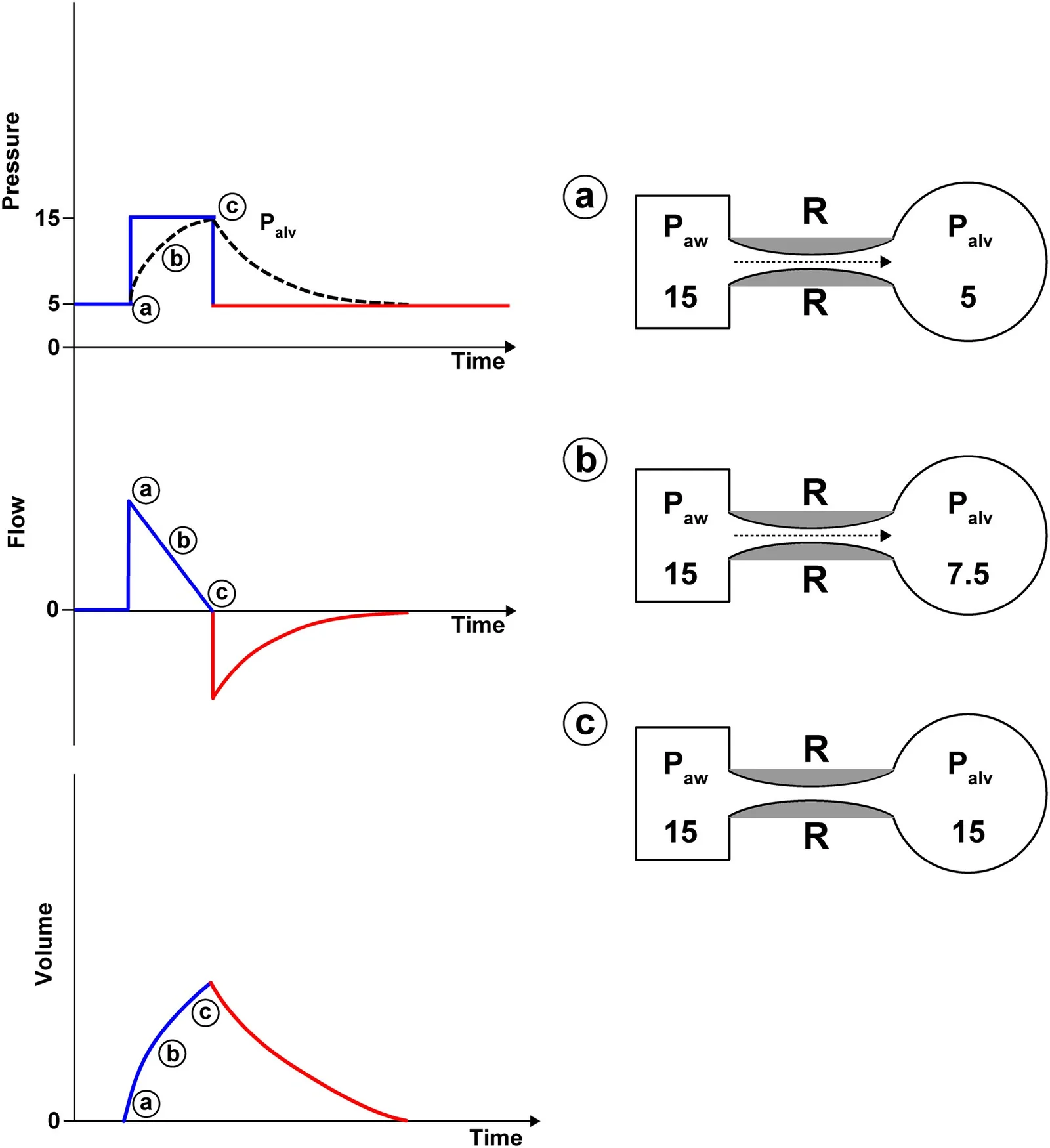
Illustration of the pressure-time, flow-time, and volume-time scalars during pressure control ventilation with a focus on passive inspiration. (A) Using an example of a patient on PC of 10 and PEEP of 5, we emphasize to the learner that, at the onset of inspiration, the pressure gradient will be largest (15-5 = 10), and inspiratory flow will be at its peak value. As air flows into the alveoli, the volume will start to increase. (B) As the alveolar volume increases, the alveolar pressure will gradually rise in an exponential fashion. As alveolar pressure rises, the pressure gradient between P_aw_ and P_alv_ decreases, and flow will correspondingly decrease. (C) The alveolar pressure will eventually equal the upstream pressure (P_aw_) at which point the flow will cease. The dotted line represents the alveolar pressure throughout the respiratory cycle, which is only measurable when the flow is paused. Abbreviations: P_alv_, alveolar pressure; P_aw_, airway pressure; PEEP, positive end expiratory pressure; R, resistance.

As air begins to fill the alveoli at a constant flow rate (ie, square-wave flow), we point out that volume will increase in a linear fashion. As a result of the linear increase in volume, P_alv_ will also increase linearly in accordance with the equation of motion. We highlight that the slope of this linear portion of the P_aw_ curve is dictated by V/C component of the equation of motion (Figure 2C). When inspiration ends and expiration begins, P_aw_ returns to PEEP_set_ and the gradient for airflow now reverses (ie, from the lungs toward the patient; Figure 2D).

We now give the learners a few minutes to draw the normal VCV curves themselves. We remind them to start with the fixed curve(s), in this case the F-time and V-time curves, which are predictable since they are being controlled. Then we remind them to identify the variable curve(s) next, in this case the P_aw_-time curve, and ask them to recall the equation that determines the variable curve, which for VCV is the equation of motion. The equation should remind the learner that there are 3 components to the P_aw_-time curve, each corresponding to the 3 components of the equation: PEEP_set_, F*R, and V/C. To facilitate interaction, we typically break the audience into groups to work together on these questions for a few minutes before having them present their drawings to the audience. We then ask them to redraw what the scalars would look like in the setting of bronchospasm (increased R) or pulmonary edema (decreased C). For example, development of pulmonary edema (decreased C) will increase the slope of the P_aw_-time and P_alv_ curves and result in a higher PIP for a given V_T_ (Figure S3). Depending on time constraints, we also use this opportunity to cover the concept of the stress index to relate how changes in respiratory system C over the course of the delivered breath may affect the morphology of the P_aw_-time curve (such as crossing the lower or upper inflection point; Figure S4).

We cover VC decelerating-flow ventilation in a similar manner; however, we emphasize that because flow is decreasing over the course of the breath, alveolar pressure increases in a nonlinear fashion and the F x R component of the curve decreases over the course of the breath (or in other words, the difference between P_aw_ and P_alv_ decreases over the course of the breath; Figure S5).

### Pressure control breath

Just as we had done for VCV, we begin by reiterating the questions above: Which curve(s) is fixed for PCV? For PCV, as the name implies, we teach that the P_aw_-time curve is fixed during inspiration over a set amount of time and therefore should not vary substantially from breath to breath. At the start of inspiration on the P_aw_-time curve, P_aw_ vertically rises rapidly from PEEP_set_ to the prescribed P_aw_, remaining constant over the set inspiratory time before cycling to expiration and dropping back down to PEEP_set_ (giving the appearance of a rectangle on the P_aw_-time curve) (Figure 3).

We then ask, “Which curve(s) is variable?” For PCV, we remind the learner that since P_aw_ is being controlled by the clinician, the F-time and V-time curves are variable. We direct the learner to the F-time curve and ask, “Looking at our equation for flow, when will flow be highest during inspiration?” They should notice that F increases rapidly at the start of inspiration because the pressure gradient for F (P_aw_-P_alv_) is highest at this time point. As air flows into the alveoli, P_alv_ rises as alveolar volume increases (P_alv_ = V/C + PEEP_total_). We will often draw an arrow pointing upward next to V from the equation and then another arrow pointing upward next to P_alv_ to emphasize that P_alv_ increases as V increases. From here, as P_alv_ rises, the pressure gradient for F decreases, and we draw a progressive decline in F over the course of the breath (Figure 3). We stress that inspiratory F will continue until P_aw_ = P_alv_ (and therefore, there is no pressure gradient), or the set inspiratory time is reached and the ventilator cycles to expiration (Figure 3). It is important to emphasize once again that the pressure-time curve is modeling P_aw_, which is set at the proximal airway near the ventilator, therefore, the rise in P_alv_ is not displayed on the scalar and occurs “behind the scenes.”

We again break up the learners into groups and give them a few minutes to draw the normal PCV curves themselves. If given the time, or at a subsequent session, we will then ask them to redraw what the scalars would look like in the setting of bronchospasm (increased R) or pulmonary edema (decreased C) (Figure S6).

### Volume targeted ventilation

For volume targeted ventilation (VTV), we explain that although the clinician “sets” the V_T_, the architecture of the breath is similar to a PCV breath, and the ventilator applies a P_aw_ above PEEP_set_ to achieve the V_T_ targeted by the clinician. Based on the V_T_ generated by these PC breaths, the ventilator will either increase or decrease P_aw_ to achieve the V_T_ targeted by the clinician. For example, if the clinician prescribes a V_T_ of 500 mL, the ventilator will deliver a PC breath to achieve this V_T_. However, if the V_T_ achieved was only 350 mL for that breath, the ventilator will then increase P_aw_ on the following breaths (and therefore the ΔP dictating airflow between ventilator and patient) to generate the targeted V_T_. The opposite would occur if the V_T_ generated were more than 500 mL, then the ventilator decreases P_aw_ on subsequent breaths in an attempt to decrease V_T_. We refer back to our equation of motion and illustrate that the only component truly clinician controlled in VTV is PEEP_set_ (Table 1B).

In our experience, because VTV essentially utilizes PC waveforms on a breath-by-breath basis, if we have already covered normal PCV waveforms, we do not need to repeat all the steps in explaining PCV waveforms again. Instead, we highlight how the P_aw_-time curves in VTV change based on variations in tidal volume, including with changes in R and C.

### Patient-ventilator interaction and pressure generated by the respiratory muscles

Up until this point, we have focused on passive scenarios in MV. However, the patient is also able to generate pressures in the respiratory system. Including pressure generated by the respiratory muscles (P_musc_) in the equation of motion, we now have:


$$P_{aw}-P_{\mathrm{musc}}=F\;x\;R+V/C+\mathrm{PEE}P_{\mathrm{total}}.$$

We emphasize that in certain clinical scenarios, patients may experience increased respiratory drive,high minute ventilation and flow demands. These scenarios may result in significant PVA such as flow/volume starvation and premature cycling. The subsequent sections of our course therefore cover the principles of abnormal waveforms, patient-ventilator interactions, and MV asynchrony. However, these concepts are beyond the scope of this article. Now that the learner has a firm understanding of normal waveforms, and more importantly, *why* they appear as they do, the ability to detect serious, and potentially life-threatening waveform patterns becomes manageable.

## Why is that the approach?

Our approach is predicated on several important educational philosophies. A fundamental aspect of the course is its use of a spiral curriculum, that is an iterative revisiting of concepts throughout the course that build on each other in a way that deepens understanding of increasingly complex material.^9^ While the concept of a spiral curriculum is often associated with more longitudinal curricula that span months to years, we incorporate several key features of the spiral curriculum in the teachings of our condensed course. First, we aim to introduce concepts in increasing levels of difficulty, starting with simple mathematical equations before advancing to normal waveforms. Second, we ensure that new topics are related and built on prior topics. For example, we teach the shape of MV waveforms using the recently learned mathematical equations of Ohm’s law and the equation of motion to inform precisely why the waveforms look the way they do. This not only provides an opportunity to revisit prior topics but also provides linkages between each lesson that help facilitate a deeper understanding of the material and increase the learner’s competence with each lecture.^10^ Along these lines, our educational approach also incorporates aspects of cognitive load theory by beginning with low complexity principles and gradually increasing the complexity of the material to avoid overloading the learners.^11^

## What challenges in teaching the material should be anticipated?

While learners may have previously had significant exposure to concepts in MV surrounding basic modes, adjustment of ventilator settings to achieve adequate gas exchange and the basics of lung protective ventilation strategies, several of the concepts we introduce in this article may be unfamiliar to the learner. The equation of motion may be daunting to learners in the beginning; however, we find that systematically illustrating how the equation is derived from Ohm’s law and combined with corresponding figures, including diagrams (eg, 2 balloons connected by a tube), idealized waveforms, and real-life ventilator waveform pictures, allows most learners to understand the topic. Learners may become confused about the difference between P_aw_ and P_alv_, and the educator may have to frequently emphasize the difference, often by pointing to the aforementioned model figures. Educators may face time constraints in presenting the material, especially outside of a dedicated classroom setting, such as in the ICU. Therefore, it is vital to carefully plan how to chunk the lectures appropriately so they can be presented in a piecemeal, iterative process.

Author contributions

M.K., N.S., and B.L. each contributed to the writing of this manuscript.

## Supplementary Material

aapag020_Supplementary_Data
