# Supplementary material for: How I Teach Mechanical Ventilator Waveform Analysis: A Focus on Normal Ventilator Waveforms
Source: ATS Sch. 2026 Apr 20;7(3):233–40. doi: 10.1093/atsscholar/aapag020 (PMC13519319; doi:10.1093/atsscholar/aapag020)
Supplement: Supplementary file 1 [file aapag020_supplementary_data.zip › HIT - Normal Waveforms Supplementary Index Clean Version.docx]

**Supplementary Index**

**PIP (Paw = F x R + V/C +PEEP_total_)**

**Palv (V/C +PEEP_total_)**

**Flow is paused during inspiratory hold maneuver**

**Supplementary Figure 1:** Performing an inspiratory pause maneuver (momentarily making flow zero) to reveal the difference between PIP (highest P_aw_ during inspiration) and P_Plat_ (P_alv_ at the end of inspiration). We emphasize that the plateau pressure must actually plateau as in the example above to provide an accurate value. PIP: Peak Inspiratory Pressure; P_plat_: Plateau Pressure; P_aw_: Airway Pressure; P_alv_: Alveolar Pressure; F: Flow; R: Resistance; C: Compliance; V: Volume; PEEP: Positive End Expiratory Pressure.

**Supplementary Figure 2:** Above is an example of a group exercise we use to practically apply the Equation of Motion to the evaluation of changes in PIP and P_plat_. For example, in scenario A, the PIP has increased, however, P_plat_ has not changed. By scanning the Equation of Motion (P_aw_ = F x R + V/C + PEEP_total_), the learner can appreciate that because the alveolar pressure has not been affected (V/C+PEEP), and the ventilator flow settings have not changed, the reason for the elevated PIP must be an increase in resistance(R). For scenario B, P_aw_ has increased and is driven by an increase in P_plat_ (the static P_alv_ at end inspiration). The learner should recognize that P_alv_ can be affected by changes in V, C or PEEP. Because the TV settings or the PEEP_set_ have not changed, this is likely driven by a decrease in C or potentially auto-PEEP. Because the numerical difference between P_aw_ and P_alv_ has not changed, the F x R component is unlikely to have changed. For scenario C, the decrease in P_aw_ and inability to record a static P_plat_ can be explained by an air leak as volume continuously leaks out of the system. In scenario D, the PIP and P_plat_ remain the same. Therefore, there is unlikely to be any changes in the components of the Equation of Motion. This may occur in the setting of a pulmonary embolism or an extrapulmonary process such as a myocardial infarction or sepsis. PIP: Peak Inspiratory Pressure; P_plat_: Plateau Pressure; P_aw_: Airway Pressure; P_alv_: Alveolar Pressure; F: Flow; R: Resistance; C: Compliance; V: Volume; PEEP: Positive End Expiratory Pressure.

**Supplementary Figure 3:** How changes in respiratory system resistance (R) and compliance (C) alter the shape of the scalars in volume control ventilation. During inspiration, the F-time and V-time waveforms are fixed by the clinician, and therefore, should show little change. However, the P_aw_-time curve is variable and will change with changes in R and C. With increased R, the F * R component of the curve will change, represented by an increase in the height of the initial pressure increase at the start of inspiration and higher PIP (superimposed blue line). The shape of the P_alv_-time curve will remain unaffected, and therefore, the P_plat_ will remain unchanged. With a decrease in respiratory system C, the initial F * R component of the curve will not be affected, however, there will be an increased slope of the V/C portion of both the P_aw_-time (blue line) and P_alv_-time (red line) curves. Therefore, there is an increase in both PIP and P_plat_. PIP: Peak Inspiratory Pressure; P_plat_: Plateau Pressure; P_aw_: Airway Pressure; P_alv_: Alveolar Pressure; F: Flow; R: Resistance; C: Compliance; V: Volume; PEEP: Positive End Expiratory Pressure.

Violate UIP

End-inspiration

End-expiration

[Grab your reader’s attention with a great quote from the document or use this space to emphasize a key point. To place this text box anywhere on the page, just drag it.]

Violate LIP

**Supplementary Figure 4: The Stress Index:** We emphasize to the learners that if compliance of the respiratory system changes over the course of the delivered breath, such as if the patient were to cross the upper or lower inflection point on their static pressure-volume curve, the slope of the P_aw_ curve will no longer be linear and change over the course of the breath. For example, if the upper inflection point (UIP) is being traversed, compliance will decrease over the course of the breath resulting in an increase in slope of the P_aw_-time curve over the course of the breath. The opposite phenomenon occurs when crossing the lower inflection point (LIP). P_aw_: Airway Pressure; F: Flow; R: Resistance; C: Compliance; V: Volume; PEEP: Positive End Expiratory Pressure.

**Supplementary Figure 5:** P_aw_-time, F-time and V-time waveforms for a normal, passive VCV decelerating flow ramp breath. As F decreases throughout inspiration, the size of the F * R component (representing the difference between P_alv_ and P_aw_) decreases. If the F pattern decreases to zero F as it does in the breath represented here, P_aw_ will equal P_alv_ at the end of inspiration. Because F decreases over the course of the breath, the slope of the V-time and P_alv_-time curve also decreases over the course of the breath. This contrasts with square wave flow VCV, in which constant F over the course of the breath results in a linear rise in V (and P_alv_), and the difference between P_aw_ and P_alv_ should not substantially change throughout the breath because F is constant (assuming there is little change in R throughout the delivered V_T_). P_aw_: Airway Pressure; P_alv_: Alveolar Pressure; F: Flow; R: Resistance.

**Supplementary Figure 6: Pressure Control breaths with changes in resistance and compliance:** We emphasize that, in contrast to VCV, where changes in R and C affect the P_aw_-time curve, during PCV, changes in R and C of the respiratory system will result in changes in the F-time (and V-time) curve. We illustrate that, all other things equal, according to Ohm’s law (and the Equation of Motion), an increase in R will result in lower inspiratory flow rates at any given point in time. As a result of these lower flow rates, the rise in alveolar volume, and therefore the rise in P_alv_, will be slower. In this setting, the rise in P_alv_ may be slow enough that P_alv_ never reaches P_aw_ and F persists throughout inspiration. The opposite occurs with a decrease in R. F at any given timepoint is higher and P_alv_ rises at a more rapid rate, potentially equilibrating with P_aw_ earlier, with cessation of F throughout the remainder of the inspiratory time. A decrease in respiratory system C will result in a more rapid increase in P_alv_ over the course of the breath (greater change in pressure for a given volume). As a result, F decreases more rapidly from peak inspiratory flow to zero. P_aw_: Airway Pressure; P_alv_: Alveolar Pressure; F: Flow; R: Resistance; C: Compliance; V: Volume.
